# Midwifery as an Occupation and Identity in Jennifer Worth’s *Call the Midwife*

**DOI:** 10.1007/s10912-025-09938-5

**Published:** 2025-04-03

**Authors:** Zlatina Nikolova

**Affiliations:** Department of Communications, Drama and Film, University of Exeter, BG.24, Queen’s Building, The Queen’s Drive, Exeter, Devon EX4 4QH UK

**Keywords:** Midwife, Femininity, Identity, Tools, Occupation, Thing theory, Post-war London

## Abstract

An autobiographical account of Jennifer Worth’s life as a midwife in the East End of the 1950s, *Call the Midwife* (2002), explores a world populated largely by women. Worth’s stories of motherhood’s anxiety, pain, joy, and occasionally unspeakable grief are underscored by descriptions of the tools and surgical procedures performed by the dedicated midwives of Nonnatus House. This essay reflects on the construction of the figure of the midwife through the materiality of the objects and tools of her occupation, and the performance of surgical procedures. Worth’s accounts of medical procedures or the use of tools establish the individuals of her narrative as midwives first, and as women second. In the eyes of everyone: mothers, fathers, and society as a whole, the midwife is defined by her profession, from her distinctive uniform to her skillset and tools, and by her commitment to her community. Drawing on the history of midwifery, thing theory, and the broader contexts of post-World War II London, this essay analyses Worth’s text in relation to questions of female identity and thing theory.

In the first chapter of her memoir *Call the Midwife* (2002), Jennifer Worth interrupts an account of a night-time birth to pose the question, ‘Why aren’t midwives the heroines of society they should be?’ (2002, 16). Charged with the urgency of labour and new life emerging, Worth’s poignant query is reflexive of the unappreciated specialist knowledge and silent moral support midwives historically lent to their communities. Worth’s remark can be situated in the broader historical and institutional contexts of midwifery’s evolution from a manual occupation, unacknowledged by the medical profession, to a discipline in its own right. Originally neglected because of its focus on the female body, midwifery’s occupational progress is intertwined with the invention of the tools and techniques of obstetrics, leading to the subsequent subversion of the midwife’s skills by other members of the medical community and her specialism’s complicated relationship with the objects and processes of its practice. As the midwife works in the crucial moments of Muriel’s labour, Worth lists her uncomplicated toolkit, including ‘scissors, cord clamps, cord tape, foetal stethoscope, kidney dishes, gauze and cotton swabs, artery forceps’ (2002, 12). The midwife does not require complex equipment to perform her duty; instead, her tools are lightweight and easily transported via bicycle at any hour, hinting at the kinetic urgency that defines her profession (Worth 2002, 12). The inventory of the maternity pack collected by Muriel’s husband earlier is equally simple: maternity pads, disposable absorbent sheets, and brown paper, which, though old-fashioned, Worth informs us, is convenient for discarding the used equipment after the birth (2002, 12). This brief account juxtaposes the simplicity of the midwife’s tools and artefacts with the complexity of her work and the proficiency of her skill, establishing this essay’s interest in the agentic materiality of midwifery and the ways the objects of the profession encapsulate its history and the midwife’s identity.

Drawing on the historical evolution of midwifery practice and the contexts and approaches developed through thing theory, this essay reflects on the role of medical tools and practices in the depiction of the midwives in Worth’s *Call the Midwife*. Part autobiographical reflection, part historical inquiry, Worth’s memoir has also been adapted into a successful BBC TV series that has been ongoing since 2012, further illustrating the broader interest in midwifery as an occupation and this particular historical moment of its development. Rather than the dramatisation of the midwives’ personal experiences, however, the original text of *Call the Midwife* is geared towards an interrogation of the social, historical, and scientific circumstances of the profession. The text abounds in recollections of processes and tools, as well as short descriptions that position its narrative in the moment following the gradual absorption of midwifery into the academic structures of medical education, all underscored by the intimacy of Worth’s first-person narration as she reminisces the details of being a midwife. The episodes of childbirth scattered in-between the records of the midwives’ daily routines juxtapose the modesty of the midwife’s equipment with the scientific intricacies of her mission to bring children into the world, foregrounding her technical expertise and her moral duty to the mothers she aids.

Apart from illustrating the midwives’ technical proficiency, the text’s emphasis on the materiality of their occupation structures them as people. The midwives of *Call the Midwife* trade their own femininity in favour of their devotion to their specialism, condensing their personalities in the functionality of their tools. Placing their patients’ needs and the demands of their calling first, their identities are encapsulated within midwifery’s toolkit and its place in the medical hierarchies, erasing the traces of their intimate lives. Worth extends the same significance of objects to the rest of the individuals outside the small cast of midwives in Nonnatus House, as the community’s existence is shaped by its material circumstances. While this association between people and objects reinforces the significance of occupational and other artefacts for the text’s formal structures, it also reveals the environment in which the young midwives operate. As they make allowances for the imperfect living conditions of London’s East End, the midwives are brought closer to the communities they serve as the text humanises their efforts. This not only sets them apart from doctors and other medical professions but also places them at odds with the indifference of the 1950s’ medical institutions as a whole.

## The materiality of a profession 

The connection between midwifery and its tools is not singular within the evolution of medical disciplines and can be related to the broader critical contexts of thing theory, an alternative approach to the study of artefacts, and everyday objects in the 1980s (Miller 2002 [1998], 3). David Miller’s introduction to the volume *Material Culture* clearly separates thing theory from other existing studies of consumerism and commodity culture (2002 [1998], 3–21). Rather than focusing on commodities and consumerism, thing theory proposes a discursive model where the corporeality of different objects is employed as an analytical framework for the ‘fabric of cultural worlds’, signposting such artefacts’ potential to define and even conceive social, occupational, cultural, and other hierarchies (Miller 2002 [1998], 6). Drawing on their ability to signify various areas of activity and society, objects encapsulate multiple dimensions of one’s identity (Miller 2002 [1998], 6). Jennifer Sattaur develops Miller’s claims by proposing that by emphasising objects’ material significance, thing theory adopts cultural materialist and historicist approaches more akin to literary and historical scholarship (2012, 347). Sattaur’s qualification of thing theory can be traced through similar subsequent texts that imagine artefacts as more than possessions, positing them as signifiers of the more abstract networks that govern contemporary society. In their volume *The World of Goods*, Mary Douglas and Baron Isherwood conduct an anthropological study of the interconnections between consumption and identity (2021/1979). In her volume *The Social Psychology of Material Possessions*, Helga Dittmar builds Isherwood and Douglas’ ideas by indicating a deeper correlation between one’s identity and material possessions’ symbolism of their class, qualities, and personal attitudes (1992, 63, 125). An embodiment of social relationships and perceptions of the individual self, the ownership of objects allows one to exert control over their environment or exercise their authority and power within social hierarchies (Dittmar 1992, 52, 56). Extending the discussion of objects’ agentic qualities in shaping identity, Vera Mark writes about the connection between artefacts and their creator, and the ways the objects, their surfaces, and their processes of production may even encode political meaning (1994, 63–100).

Similarly exposing the intertextual ‘semiotic web’ of objects’ role in everyday life and culture, Stephen Harold Riggins recognises the ‘socialness’ of artefacts and their active contribution to the formation of social dynamics (1994, 3). Riggins’ interpretation of objects’ functions for the individual can be examined in conjunction with Miller’s claim that material things are both visible and invisible (1987, 108), highlighting objects’ overt applications and covert influence within social networks and identity creation. The same suggestion about the obvious functions of objects and their broader significance is laid out in Riggins’ model of objects as participating in complex relationships of perpetual meaning-production and signification through their physical properties, the space, and other artefacts that surround or interact with them, people’s experience and use of similar items, and texts about these objects (1994, 3). This approach not only endows material items with symbolism but extends its influence further beyond their physical boundaries and into the practices of their production and subsequent application. Thing theory’s postulation of material possessions as the source of identity creation and social and historical contexts can be extended to the more specialised study of occupational materiality and professional identity, such as this essay’s interest in the tools of midwifery and its hierarchies.

Although not informed by the principles of consumerism and commodification inherent in capitalist society, medical history is punctuated by the discovery or innovation of processes, tools, and objects that define professional identities across its hierarchies in similar vein to the theories mapped out by Dittmar, Riggins, and Miller. Processes and tools are thus intertwined with the structures of the medical field, establishing objects just as significant as scientific techniques. Tools allow medical professionals to perform certain roles, as they are tailored to medical disciplines, methods, processes, and even to specific actions within complex procedures. Thus, the material properties of medical tools allow them to shape certain medical disciplines. Existing historical texts on different aspects of medicine testify to this: Arnold van de Laar’s *Under the Knife* (2018) traverses history highlighting the origins of surgical tools, while Lindsey Fitzharris’s *The Butchering Art* (2017) delves into Victorian medicine, studying the contributions of surgeon Joseph Lister and the procedures he trialed and developed. These historical connections respond to Riggins’ suggestion that artefacts are semiotically charged by the spaces and contexts they inhabit, containing information beyond their immediate applications (1994, 3). This correlation between the diversity of medical disciplines and their respective equipment illustrates the convergence between texts, spaces, and artefacts’ functions that Riggins recognises. The association between objects and the individuated human is further developed by Arjun Appadurai (2013, 3–63), one of the foundational contributors of thing theory.

Appadurai suggests that while ‘from a *theoretical* point of view human actors encode things with significance, from a *methodological* point of view it is the things-in-motion that illuminate their human and social context’ (2013, 5). In an illustration of the complex dynamics of human-object relations, Appadurai’s emphasis on the methodological role of things is particularly applicable to medical contexts and their disciplinary dedication to certain toolkits and technical procedures as it extends the function of objects beyond identity formation and into occupational contexts. Appadurai’s implied proposition is that the roles humans assign to objects are less significant than what actions objects allow humans to perform and the contexts in which they are used. Appadurai accentuates the hold that objects have on various activities and occupations, justifying the material multiplicity of the world from the early twentieth century onwards. Bill Brown’s addition to thing theory further tries to make sense of the preponderance of stuff that characterises contemporary life (2004). Brown differentiates between things and objects by proposing that ‘we look *through* objects… but we only catch a glimpse of things’ (2004, 4). We examine objects because they are discursively encoded as information carriers as opposed to things, which can be easily discarded once they are no longer of use, and have lost their significance and function (Brown 2004, 4). Building on Appadurai’s methodological suggestion in an attempt to understand the human-object relations of the contemporary material world, Brown positions inanimate objects and their ability to perform certain functions as constitutive of human subjects and their histories (2004, 7).

The various contributors to thing theory indicate several important parallels between objects and human occupations that can be employed as theoretical frameworks for both the histories of midwifery and Worth’s own experiences as a midwife. Thing theory’s interventions in our engagement with objects suggest firstly that objects with clear functions carry specific discursive significance that can be read and analysed, rendering these objects into agents of historical and personal narratives; secondly, that separate professions are interconnected with specialised objects with singular functions to the extent that these become definitive and exclusive to the occupations and the disciplines that utilise them; and thirdly, that objects and their functions can be illustrative of the individuated humans who employ said objects.

## Artefacts’ instrumentality to the development of midwifery

The correlation between tools and professional practice, on the one hand, and the individual and their occupation, on the other, indicated by the various articulations of thing theory examined in the previous section, are illustrated across the history of midwifery in Britain. Under the governance of a range of external authorities including general practitioners, hospitals, and subsequently, the National Health Service (NHS), midwifery evolved from a non-technical manual occupation delegated to non-academically educated women from within the community into an academic discipline that required years of training and practice. In midwifery, this essay sees an example of the ways objects shape professions and professional identity as per Appadurai’s take on thing theory (2013, 5), and an illustration of tools and artefacts’ ability to introduce social hierarchies and tensions, as in Dittmar’s writing (1992).

Unlike other medical professions, midwifery’s adoption of its specialist instrumentality is gradual. Tracing midwifery’s origins from the medieval period to the twentieth century, Marjorie Tew highlights the midwife’s initial non-interventionist philosophy and dedication to the natural process of childbirth (1995, 9). The midwives’ relationships with the community they served and their non-academic medical status juxtapose them to the educated physician. Under the influence of obstetricians in later centuries, however, the integrity of the midwives’ manual and non-technical methods was compromised, and they were consequently trained to perform certain minor interventions that did not require specialist equipment (Tew 1995, 9). Had they been allowed to acquire more technical proficiency and more tools, they would have rivalled the academically educated obstetricians themselves, and that had to be avoided (Tew 1995, 9). As the essay’s exploration demonstrates, the more midwifery’s instrumental materiality expanded, the more it shaped the profession by diminishing the authority of the singular midwife and passing it on to the male authority of the physician, general practitioner, or to any of the relevant institutions of healthcare. However, the midwife’s use of specific tools and her highly specialised knowledge also endowed her with a peculiar kind of authority over the feminine realm of motherhood, transforming her into an inseparable member of local communities that other medical professionals and formalised healthcare services struggled to replace.

With time, midwifery became subject to increased professionalisation and institutional interference in Britain: first, by the Church (Allison 2021, 60–62; Moscucci 1990, 43–44); and later, by organisations such as the Royal College of Physicians, the Company of Surgeons, and even the Society of Apothecaries (Moscucci 1990, 52–53). An attempt to establish a similar institution dedicated to midwifery was hampered by the Royal College of Physicians (Wilson 1995, 32). Regardless of this tripartite organisation of the medical professions and the internal tensions over their disciplinary scope, people were rarely able to afford professional physicians’ help and sought medical advice elsewhere (Moscucci 1990, 52–53). Surgeons’ daring determination to expand their practice by entering midwifery therefore did not infringe upon anyone else’s practice but that of the midwife who was unprotected by such institutions.

While some of the historical texts referenced here imply that the midwife’s approach left the process of birth to the laws of nature, historical accounts of midwifery practices by Anne Borsay and Billie Hunter (2012) and studies on the rise of man-midwifery by Adrian Wilson (1995) assert that midwives actually commanded sufficient authority in their communities and employed artefacts and processes of their own. These discoveries point not only to midwifery’s complex history but also to the correlation between the field’s specificity and its own instrumentality. Although formal medical education was out of reach for them, the midwives came from learned and stable families, and were just as knowledgeable as any doctor until at least the early eighteenth century (Cody 2005, 35). Midwives of the early eighteenth century still performed certain procedures such as turning of the foetus, and they prescribed medications and employed tools such as crotchets and hooks, and occasionally the fillet, one of the tools developed by the Chamberlen family (Borsay and Hunter 2012, 109; Wilson 1995, 65).

While midwifery itself was considered a manual occupation, unsuitable for the academically educated physicians, it was also seen as a necessary component to a successful general practice, an initiative that posed problems for the midwives (Borsay and Hunter 2012, 130). Physicians’ reluctant acceptance that midwifery should be part of their own skillset put the start to the broader tensions between midwives and the medical profession, and forced midwifery’s increased specialisation as a discipline. Physicians’ or surgeons’ approaches towards labour and childbirth were different to the naturalistic and humane practices of the midwife. Male medical professionals’ ignorance was rooted in scientific confusion as well as the general social attitudes that pregnancy and birth were women’s business, and therefore only someone who had experienced childbirth themselves could comprehend the ways of a woman’s body (Cody 2005, 35–36). Male medics’ marginal understanding of the female body and its processes led to the construction of various models and obstetrical machines, included in the training of men-midwives and other male representatives of the medical professions (Lieske in Mangham and Depledge 2011, 69–88). The complexity of the female body and the uterus resulted in the creation of obstetrical machines and their wide use across the medical profession, as Pam Lieske recounts (Lieske in Mangham and Depledge 2011, 69–88). Ranging from wax models to be exhibited to the public and scientists alike, to sophisticated apparatuses or manikins, devised by men, these models were incorporated in the training of men-midwives as it became subject to formalised certification (Lieske in Mangham and Depledge 2011, 71). In yet another instance of reshaping midwifery through material objects and tools, these models laid bare the intricacies of the female body and specifically, women’s reproductive systems, subjecting them to the gruesome curiosities of male doctors and the wider society. The models became objects of detailed study and attention as the midwives’ male counterparts struggled with what they had not and could not experience, underscoring the gendered aspects of childbirth and of the midwife’s profession. These developments problematised the position of the midwife in her medical and social networks by dismissing her expertise while allowing her to remain necessary because of her gender-specific occupation. In essence, the increased use of medical objects and tools, and the development of more complicated practices of delivery compromised the figure of the midwife and eclipsed the status and value of her occupational proficiency. Therefore, the development of more scientific expertise was out of grasp for the midwife for a long time, until midwifery as an occupation was admitted to the halls of medical education.

In the second half of the twentieth century, the formation of the NHS identified the general practitioner as the main contact for pregnant women, mothers, and their children, promoting a further shift from home births to hospital births (Borsay and Hunter 2012, 157). This institutional repositioning of responsibility prompted anxiety in communities who distrusted the authority of the hospital and its impersonal sanitised spaces, as Worth describes in the chapter dedicated to Sally and her own recollections of historical treatments of eclampsia (2002, 81–82). Regardless of her doctor’s attempts to convince her otherwise, Sally approaches the midwives of Nonnatus House out of fear of lying-in infirmaries, and following advice from her mother (Worth 2002, 81). In her account of this episode and its historical contexts, Worth notes that this transition to hospital births further contributed to the marginality of the district midwife, who slowly became an ‘extinct species’ (2002, 81). Although brief, this episode’s references to the changing social and professional contexts of obstetrics hint at the importance of the environment and the objects of care specific to the midwife’s occupation. As the environment in which she operated started to change, her function in her community began to diminish.

Thus, the clear delegation of care-giving and expertise provoked by institutions and evolving socio-historical contexts reaffirms the material interconnections between objects and their humans noted by both Brown and Appadurai. Each of the care-giving roles mentioned in these histories is shaped by its tools, exemplifying Appadurai’s methodological approach towards objects and their discursive significance as highlighted by Brown. However, the instrumentality of these occupations and the spaces they employ for their care-giving is further complicated by the symbolism of the divergent scientific and social responsibilities encoded in each medical specialism. It is also this historical moment and professional hierarchies that *Call the Midwife* illuminates in its depiction of midwifery.

## Artefacts, tools, and professional identity in *Call the midwife*

The semiotic functions that thing theory assigns to artefacts are exemplified in both objects’ encapsulation of occupational identity across *Call the Midwife* and in the ability of the midwifery toolkit to establish the broader social spaces and historical contexts of the profession. Devoted to a closer study of Worth’s text, the following section reflects on the use of objects and specialist artefacts in the urgent moments of the midwife’s work, the text’s own literary depictions of the midwives and those around them, and finally, the social and historical spaces and contexts in which the midwives operate. As the midwives sacrifice their individuality to their occupational commitments, they become an assemblage of the objects and practices of their work, signalling the intricate network of artefacts that determines their profession and their position in the broader social and medical hierarchies.

The text’s opening chapter not only illustrates the simplicity of the toolkit on which the midwife and the mother and child depend but also juxtaposes the significance of new life emerging to the scientific process this entails. Even as the mother dotes on her newborn, the midwife continues her work as she waits for the placenta to be expelled from the uterus (Worth 2002, 15–17). The contrast between the tender first moments of a mother’s love for her newborn and the midwife’s medical proficiency is achieved even on linguistic level, as Worth’s description of the process is packed with medical and anatomical terminology:

I massage the fundus in my hand until it is hard and round and mobile. Then, I grasp it firmly, and push downwards and backwards into the pelvis. As I push, the placenta appears at the vulva, and I lift it out with my other hand. The membranes slide out, followed by a gush of fresh blood and some clotted blood (2002, 17).

The combination between the text’s autobiographical delivery and its insertion of technical language accentuates the midwife’s concentration and devotion to her patient, as well as her knowledge and ability to perform complex processes with a limited toolkit. The shift from past to present tense, a frequent stylistic trait of Worth’s writing across this text, imbues the moment with urgency despite the emotional charge of newborn life. Worth’s explanation that in later years the same process would be aided by oxytocics further consolidates the midwife’s knowledge and the exiguity of her tools under the institutional limitations of her activities (2002, 17).

The complex and occasionally perilous nature of the processes that Worth describes foregrounds the midwife’s knowledge against the historically ignorant assumptions surrounding women’s health and their reproductive faculties (Cleghorn 2021). One such instance is Worth’s account of a breech delivery. The episode involves three of the characters: Worth herself as a midwife still in training, Sister Bernadette, one of the sisters from Nonnatus House, and the local GP, Dr Turner. As the young midwife and Sister Bernadette instruct the mother, the general practitioner Dr Turner is called to aid the birth following the discovery of the complications (Worth 2002, 104). In recording the interactions between the two midwives and the GP, Worth’s memoir takes advantage of the moment in order to once again highlight the midwives’ skills and juxtapose the ingenuity of their technical expertise to the doctor’s bureaucratic superiority, reinforcing the social and hierarchical inequalities of their professions. With the doctor’s approval, Sister Bernadette is entrusted to deliver the child, employing the Mauriceau–Smellie–Veit manoeuvre and the speculum (Worth 2002, 104–108).

The majority of the chapter here is taken up by the careful account of the technical process and the use of the tool as Dr Turner retreats, allowing Sister Bernadette to take control. Worth writes how everyone ‘gowned, masked and scrubbed up again’ as if listing the tasks on a scientific sheet (2002, 104). This style of scientific account continues, as she notes that the ‘perineum continued to distend’ (Worth 2002, 105). Sister Bernadette’s measured remarks punctuate the text’s description, as she recounts her method for the young midwife and for the reader, and justifies her decisions: ‘The baby’s legs will be curled up. I will want to rotate the baby to ensure the best position for delivery, but also the pull of gravity as the baby’s body hangs… This will be important’ and ‘This is the correct thing to do’ (Worth 2002, 105–106). Here, the text picks up on the shift from past to present tense in the form of Sister Bernadette’s careful commentary of the procedure in language suggestive of Worth’s own account of the technique. The detail and authority of Sister Bernadette’s commentary demonstrates the midwife’s knowledge and illustrates her humane and personal approach to the delivery, depicting her as a scientific figure endowed with both responsibility and trust. While she explains her decisions, Sister Bernadette instructs the mother: ‘I want you, Betty, to push now with all your strength…’ and ‘Now push… as hard as possible’ (Worth 2002, 106), once again signalling her calm proficiency and the respect she commands. Sister Bernadette’s guidance and actions, and Worth’s assistance, transform the midwives into the scientific conduits of a natural process as they guide the mother and child through the moment. Verbs such as ‘inserted’, ‘hooked’, and ‘rotating’ describe Sister Bernadette’s deft manipulation of the baby’s buttocks with her hands as if she is the tool herself (Worth 2002, 105). The text intersperses Worth’s remarks on her anxiety over the danger of the moment as she holds out the baby to hang unsupported (2002, 107). However, there is no place for doubts in these crucial seconds. In such urgent moments where the text turns to technical descriptions, the midwives become tools charged with technical knowledge themselves, as their own emotional labour remains unspoken.

While the midwife cares for the baby afterwards, Dr Turner prescribes oxytocics to the mother, an infrequent practice at the time, Worth notes (2002, 108). The remark on the prescription, however, once again enforces the contexts in which the midwives operate and the limitations imposed on their occupation as their access to items and practices that other medical professionals can employ is withheld. Dr Turner’s sporadic and minimal involvement in this episode contrasts Sister Bernadette’s technical performance of the delivery, signalling the social and professional distance between him and the mother, as well as him and the midwives whose participation in their community necessitates both technical skill and emotional labour. Although the GP exerts a level of institutional authority that ensures his access to medication, he is removed from the physicality of the midwife’s work processes. Nevertheless, even in the 1950s, as Worth celebrates midwifery training as an assertion of the occupation’s own independence and its contribution to the well-being of its patients, she and the other midwives of Nonnatus House are reminded of their subordination to the general practitioner in the complicated networks of healthcare. While Worth’s remark that midwifery should be taught to GPs earlier in the text highlights doctors’ insufficient understanding of the discipline, their presence is still necessary in urgent situations such as the one described in this episode, even though the midwives themselves are sufficiently equipped to proceed on their own (2002, 16).

Apart from their role in Worth’s analysis of the history of midwifery, the midwife’s tools have additional significance in the formal textual frameworks of *Call the Midwife*. Once again illustrating the interplay between humans and functional objects noted by Appadurai and Brown, as well as the artefacts’ role in constructing social identity as proposed by Dittmar, Worth’s memoir fosters the midwives’ items and uniforms as signifiers of their professional devotion. The midwife becomes a figure constructed through her dependence on the objects that enable her to perform her duties. In the historical and professional contexts recorded in *Call the Midwife*, the midwife’s uniform, her bag, and her bicycle are metonymic of the individuals themselves and their professional identities. In the very first chapter, as she travels to Muriel during the night, Worth recalls: ‘In the dark I wrench at a bicycle and crack my shin. Through blind force of habit, I fit my delivery bag on to the bicycle, and push it out into the deserted street’ (2002, 9). The references to habitual routines and the use of verbs such as ‘wrench’ and ‘push’ accentuate the physicality of the midwife’s work who cycles along deserted streets and past tenements on her way to a mother in need (Worth 2002, 9–10). The expression ‘blind force of habit’ presupposes the muscle memory of a practice inscribed onto the body’s mechanisms, alongside the profession’s intellectual and scientific rigour (Worth 2002, 9). Worth’s recollections of her choice to become a midwife evoke the figure of the nurse and midwife alongside that of the policeman of the East End, inscribing her into the characteristic landscape of this milieu (2002, 10). Pedalling through the night, Worth dismisses the gendered perceptions of her uniform as a ‘sexy’ attire constructed of ‘cuffs and ruffs’, ‘pert caps’, and ‘pinched-in waists’ in favour of the utility of her ‘navy gabardine’ (2002, 9). Her rejection of such sexualisation of the uniform detaches the possibility of gendered objectification from her commitment to her work and the community she serves. Instead, the femininity of her work lies elsewhere: in the privacy of the maternal microcosm that she facilitates, aids, and preserves.

Another example of an individual structured by the objects of their occupation is Chummy, who arrives early on during Lee’s tenancy at Nonnatus House. Taunted for her ‘County type’ background and her ‘carthorse’ appearance throughout the years of her training, Chummy is depicted as someone who finds her true calling, first in nursing at St Thomas’ Hospital in London, and then subsequently in midwifery at Nonnatus House (Worth 2002, 43–44). Chummy’s path to midwifery is defined by professional and academic achievements. In yet another instance where the personal makes way for the occupational, the text explains that Chummy’s usual clumsiness vanishes in a hospital setting: ‘On the wards she never dropped or broke a thing, never moved awkwardly or crashed into things’ (Worth 2002, 43). In consequence, she remains ‘ill-adapted’ to social life but is skilled and accepted in her chosen profession, offering another example of the ways the occupational replaces the individualistic in Worth’s memoir (2002, 44). In a chapter dedicated to Chummy’s arrival at Nonnatus House, Worth describes how the enthusiastic nurse forges her identity as a midwife by tailoring her clothing and adapting to the objects and tools that encapsulate midwifery’s function in the community (2002, 41–46). Reinforcing her efficiency and commitment, the text tells us that ‘Material was obtained and, in no time at all, a couple of dresses were made’ (Worth 2002, 44). Having never learned to ride a bicycle, she finds herself having to quickly master this non-scientific but necessary skill in order to complete her runs around the East End (Worth 2002, 45–46). The connection between the midwife’s work and such objects is so great that the text suggests that Chummy’s inability to cycle jeopardises her future at Nonnatus House as Sister Juliene doubts she would learn (Worth 2002, 58). Such descriptions of physical effort external to the midwives’ strictly medical duties reiterate the text’s evocation of the overwhelming kineticism that typifies the nurses’ commitment to their work.

Worth’s accounts employ further elements of the sensorial to relate the midwives’ working conditions beyond her descriptions of medical procedures and specialist tools. Across the text of *Call the Midwife*, the midwives’ efforts are often hindered by their patients’ hygiene and general ignorance. The midwives’ proximity to the physiological and bodily dimensions of human life is demonstrated in Worth’s memories of the antenatal clinic in particular, as the midwife’s activities are detached from the sanctity of motherhood and plunged into the corporeality of the impoverished lives of the East End. Admitting that the antenatal clinic was an aspect of her job that she disliked, Worth recounts the nauseating bodily smells and the devastating impact poor hygiene and negligence could have on both mother and child (2002, 62). Her descriptions of ‘the pulsating warmth and humidity’, ‘the endless chatter’, and ‘the nauseating smells of vaginal discharge, urine, stale sweat, unwashed clothes’ reinforce these sensorial and even odious elements as essential components of the midwife’s working environment (Worth 2002, 62). In these passages of the text, the human bodies of the mothers themselves become objects of ‘unwashed female flesh’ to be inspected, cleaned, and manipulated (Worth 2002, 62). Their smells linger on the midwife’s clothes as occupational reminders that she needs to overcome, privileging her responsibilities to her patients (Worth 2002, 62). As the midwife struggles with her own aversion to these elements of the tangibly physiological nature of her profession, her devotion immerses her in the ‘hard work’ as she looks after, directs, and manoeuvres her charges (Worth 2002, 62–63).

The origins of these conditions of questionable hygiene are deeply rooted in the materiality of the living circumstances of many of Worth’s patients, and are particularly significant of the historical moment and social milieu described in *Call the Midwife*. Paragraphs on remarkable medical developments and antenatal care are juxtaposed with the circumstances in which babies are born in this period, and the conditions suffered by some of the children or their mothers. Describing the tenements of the East End, Worth recounts that while some of them originally had indoor lavatories and running cold water installed when they had been first built, by the 1950s, these buildings and neighbourhoods were considered ‘slum areas’, overpopulated, and ridden with infectious diseases and pests (2002, 32). It is in these conditions that the midwives have to find ways to operate and support the mothers, many of whom suffer complications because of their living environment, poor hygiene, and ignorance of medical requirements. Edith, a forty-year-old mother who has lived through the Blitz with her husband, is drab in appearance and smokes while feeding her baby (Worth 2002, 33). Molly Pearce, a young girl of nineteen, who is nine months pregnant, lives in squalor with her two other children and abusive husband, disregarding her mother’s advice and Worth’s efforts as a midwife (2002, 35–37). Later chapters recount Brenda’s relentless attempts at pregnancy and birthing her own child despite her deformed skeletal frame (Worth 2002, 74–80), and finally, Len and Conchitta Warren, who still love and look after their large family in the face of continuous financial hardship (Worth 2002, 127–143). 

Although Worth concedes that many of the women of the East End take pride in their hard work and their homes, many of their limitations are products of their lack of knowledge of healthcare standards (2002, 36). The reality of the East End’s living conditions is an unwelcome challenge to the midwives’ practice and yet another signifier of the importance of objects for both the text’s account of the profession and its formal literary mechanisms.

Overwhelmed by its focus on their occupation, the text only depicts the characters in relation to their contribution to the tightly knit groups of midwives working at Nonnatus House. The narrative never dwells on their subjectivities apart from Worth’s narrative voice, which delivers all depictions, deftly prioritising her younger self’s anxieties over her work and pausing its personal remarks when a description of process or tools is necessary. The rest of the staff at Nonnatus House are depicted equally sparsely, placing greater emphasis on their responsibilities and indicating that their identities are defined and redefined through their dedication to their work. Despite the all-female cast of the convent and the relative independence the young midwives enjoy, there are few inferences to their non-professional identities and indeed their femininity. Although Worth allows a few glimpses into her youthful relationship with Jimmy and their adventure on the old London taxi, the Lady Chatterley: yet another instance where an object becomes significant to an individual beyond its purely functional applications (2002, 116–126), her narrative on femininity never digresses from its accounts of patients’ experiences and histories of midwifery practice. Even though *Call the Midwife*’s narrative of the young Irish woman Mary begins with a brief remark about Worth’s experience at the Festival Hall, the chapter abruptly turns its focus to the events that led to Mary’s arrival in London, her pregnancy and her encounter with the midwife (2002, 151–159). Forsaking the memories of her youth, Worth once again foregrounds the profession’s responsibility and position in the community as the midwives and the rest of the individuals of the narrative are able to ensure safety for the young woman. Thus, while presenting them as agents of an exclusively feminine world of motherhood and childbirth, the text does little to differentiate their individual feminine identities and their professional dedication.

While Worth’s account is immersed in the historical and scientific contexts of midwifery of the 1950s, it never makes explicit statements about gender, femininity, or the semiotics of female identity. However, femininity is ever-present throughout the narrative as Worth’s account focuses on a largely female group with a few exceptions, such as Dr Turner and Fred, suited to the text’s core concern with a singularly female profession. Nevertheless, as readers, we learn little of the midwives’ social experiences as women. This essay maintains that Worth’s *Call the Midwife* is deeply intertwined with the concept of femininity: a discourse realised not through obvious statements on womanhood but rather through the text’s engagement and knowledgeable depiction of a female-oriented branch of medicine and healthcare, its histories, and hierarchies. Worth’s memoir emphasises deeply feminine experiences, saturated not with an abstract or philosophical form of femininity but rather with the materiality of the female body and the very real consequences of its reproductive functions. It is this concern with the female microcosm and the tangible science of midwifery that informs Worth’s analysis of the discipline’s status and expertise in relation to other healthcare representatives and the larger medical hierarchies that preside over care in the historical moment of the 1950s. The text’s insistence on accurate descriptions of process and tools creates a different impression of the female body in its technical and anatomical commentary on the midwife’s activities. The female body, and more precisely the anatomical structures that facilitate reproduction and childbirth, function as objects along the midwives’ tools in Worth’s technical descriptions. This approach towards the female anatomy is unlike the historical fetishisation provoked by the obstetrical models used by men-midwives in earlier centuries. To revisit this essay’s earlier analysis of theories on objects and things, the female body in Worth’s account of midwifery becomes yet another of the profession’s tools as it is manipulated to both preserve life and extract new life. Under the skilled hands of the midwives, the female body transforms into a site of knowledge and solidarity. The chapters dedicated to descriptions of childbirth treat the female body with the calm and calculated precision that typifies the midwife’s specialist ingenuity, without sensationalising the moment of birth or elaborating on the emotional side of childbirth or motherhood. Worth’s careful anatomical and procedural descriptions encode the female body with the histories and knowledge of midwifery, turning it into another object of midwifery’s occupational materiality, working to showcase the midwives’ invaluable contribution to this specific moment of womanhood.

Therefore, the objects and processes in Worth’s text are immersed in deeper complexity. The tools and procedures Worth describes help situate her specific account of midwifery within the broader historical contexts of midwifery practice, which, by extension, is a discipline whose remit has been shaped by the equipment that has been made available to it. At the same time, her text expands its use of materiality as a discursive framework in its depiction of the rest of the East End’s milieu, further exploring the methodological connection between objects and humans. By applying these concepts to the mundane and challenging daily environment it records, the text highlights the significance that some objects can have both in its formal and discursive dimensions, and for the community it reminisces. Through the symbolism of objects, *Call the Midwife* recounts a deeply feminine world, grounded in its own materiality, and a type of professional and moral dedication where women help women, fostering a culture and a community that neither extensively trained doctors nor complicated and esteemed institutions can understand or simulate.

## Data Availability

No new data was collected in the preparation of this essay.
